# Age-related evolution of serum biochemistry and intestinal fatty acid signaling, innate immune response, and barrier function in suckling and newly weaned piglets

**DOI:** 10.1093/jas/skaf313

**Published:** 2025-09-08

**Authors:** Fitra Yosi, Kristina Hartinger, Frederike Lerch, Julia C Vötterl, Simone Koger, Suchitra Sharma, Doris Verhovsek, Barbara U Metzler-Zebeli

**Affiliations:** Centre for Veterinary Systems Transformation and Sustainability, Clinical Department for Farm Animals and Food System Science, University of Veterinary Medicine Vienna, Vienna 1210, Austria; Christian-Doppler Laboratory for Innovative Gut Health Concepts of Livestock, Institute of Animal Nutrition and Functional Plant Compounds, Centre for Animal Nutrition and Welfare, University of Veterinary Medicine Vienna, Vienna 1210, Austria; Department of Animal Science, Faculty of Agriculture, University of Sriwijaya, Palembang 30662, Indonesia; Centre for Veterinary Systems Transformation and Sustainability, Clinical Department for Farm Animals and Food System Science, University of Veterinary Medicine Vienna, Vienna 1210, Austria; Christian-Doppler Laboratory for Innovative Gut Health Concepts of Livestock, Institute of Animal Nutrition and Functional Plant Compounds, Centre for Animal Nutrition and Welfare, University of Veterinary Medicine Vienna, Vienna 1210, Austria; Centre for Veterinary Systems Transformation and Sustainability, Clinical Department for Farm Animals and Food System Science, University of Veterinary Medicine Vienna, Vienna 1210, Austria; Christian-Doppler Laboratory for Innovative Gut Health Concepts of Livestock, Institute of Animal Nutrition and Functional Plant Compounds, Centre for Animal Nutrition and Welfare, University of Veterinary Medicine Vienna, Vienna 1210, Austria; Centre for Veterinary Systems Transformation and Sustainability, Clinical Department for Farm Animals and Food System Science, University of Veterinary Medicine Vienna, Vienna 1210, Austria; Christian-Doppler Laboratory for Innovative Gut Health Concepts of Livestock, Institute of Animal Nutrition and Functional Plant Compounds, Centre for Animal Nutrition and Welfare, University of Veterinary Medicine Vienna, Vienna 1210, Austria; Christian-Doppler Laboratory for Innovative Gut Health Concepts of Livestock, Institute of Animal Nutrition and Functional Plant Compounds, Centre for Animal Nutrition and Welfare, University of Veterinary Medicine Vienna, Vienna 1210, Austria; Centre for Animal Nutrition and Welfare, Clinical Department for Farm Animals and Food System Science, University of Veterinary Medicine Vienna, Vienna 1210, Austria; Christian-Doppler Laboratory for Innovative Gut Health Concepts of Livestock, Institute of Animal Nutrition and Functional Plant Compounds, Centre for Animal Nutrition and Welfare, University of Veterinary Medicine Vienna, Vienna 1210, Austria; Centre for Animal Nutrition and Welfare, Clinical Department for Farm Animals and Food System Science, University of Veterinary Medicine Vienna, Vienna 1210, Austria; Clinical Centre for Population Medicine in Fish, Pig and Poultry, Clinical Department for Farm Animals and Food System Science, University of Veterinary Medicine Vienna, Vienna 1210, Austria; Centre for Veterinary Systems Transformation and Sustainability, Clinical Department for Farm Animals and Food System Science, University of Veterinary Medicine Vienna, Vienna 1210, Austria; Christian-Doppler Laboratory for Innovative Gut Health Concepts of Livestock, Institute of Animal Nutrition and Functional Plant Compounds, Centre for Animal Nutrition and Welfare, University of Veterinary Medicine Vienna, Vienna 1210, Austria

**Keywords:** gene expression, piglet, short-chain fatty acid, serum parameters, suckling phase, postweaning phase

## Abstract

It is helpful for diagnostic purposes to improve our current knowledge of gut development and serum biochemistry in young piglets. This study investigated serum biochemistry, gut site-specific patterns of short-chain fatty acids (SCFA), and expression of genes related to barrier function, innate immune response, antioxidative status, and sensing of fatty and bile acids in suckling and newly weaned piglets. The experiment consisted of two replicate batches with 10 litters each. Piglets could suckle freely and had access to creep feed from day of life (DoL) 3. Weaning occurred on DoL28. Blood, gastric, cecal, and colonic digesta, as well as jejunal and cecal tissue were collected on DoL3, 7, 14, 21, 28, 31, and 35 (n = 10/sex/DoL). Serum liver enzyme activities were high on DoL3 but decreased thereafter, reflecting the immature state of hepatocytes after birth (*P *< 0.05). Age-related fluctuations in serum glucose and lipids indicated changes in energy metabolism within the suckling period and lower feed intake after weaning. Short-chain fatty acids increased in cecal digesta from DoL3 to 28 (*P *< 0.05). After weaning, lower gastric and cecal SCFA on DoL31 and DoL35, respectively, versus DoL28 mirrored lower feed intake, whereas colonic SCFA increased after weaning (*P *< 0.05). Jejunal and cecal expression of free fatty acid receptors and monocarboxylate transporters changed with increasing age (*P *< 0.05). For some genes including *FFAR2* and *FFAR3* in the cecum, expression levels declined from DoL3 onwards (*P *< 0.05), indicating an inverse relationship with luminal SCFA availability. However, there was no unique jejunal and cecal expression pattern for fatty acid receptors and transporters, and pattern recognition receptors (PRR), probably corresponding to the age-related changes in their ligands. Increasing expression of anti-inflammatory *IL10* in jejunum and cecum from DoL3 to 28 may be indicative of a build-up of immune tolerance (*P *< 0.05). Postweaning expression of PRR was not increased, but reduced jejunal expression of antioxidative enzymes and increased cecal expression of proinflammatory *TNFA* combined with lower expression of *MUC2*, *OCLN*, and *ZO1* compared to the suckling phase indicated compromised gut homeostasis (*P *< 0.05). Overall, the present results show study-specific age-related patterns of genes associated with mucosal metabolite sensing or defense mechanisms in the jejunum and cecum from birth to after weaning.

## Teaser

The present results provide a comprehensive insight into the age-related development of clinical serum biochemistry, microbial metabolites, and gut mucosal recognition of microbial metabolites and antigens and barrier function in neonatal piglets from day 3 of life until one week after weaning.

## Introduction

Gut maturation is a specific and very dynamic process in newborn piglets. At this age, a piglet’s gut is very susceptible to disturbances. In order to prevent intestinal disorders in young piglets, comprehensive knowledge of physiological gut maturation is crucial. Intrinsic (e.g., genetics) and extrinsic factors (e.g., nutrients and microbes) drive the postnatal gut maturation including immune and barrier functions (Everaert et al., 2017). Besides sow milk and creep feed, the gut microbiota is a major source of fatty acids (**FA**), including short-, medium- and long-chain FA (Saika et al., 2019). The various dietary and microbial FA do not only have a nutritional role but they are also important precursors for bioactive mediators regulating inflammatory signals in the body (Metzler-Zebeli, 2021). Locally, FA trigger various host physiological processes by binding to gut mucosal receptors, such as free FA receptors (**FFAR**) and hydroxycaboxylic acid receptors (**HCAR**), or are absorbed (Kaiko and Stappenbeck, 2014). The receptors are selective for a particular carbon chain length of free FA. Free fatty acid receptor-1 and FFAR-4 are activated by medium-chain and long-chain FA, whereas FFAR-2 and FFAR-3 have high affinity for short-chain fatty acids (**SCFA**) (Kimura et al., 2020). Alterations in the microbial colonization in the first weeks of life may modulate expression levels of the various FA receptors along the intestinal tract. Monocarboxylate transporters (**MCT**) differ in their affinity to the various FA including SCFA, medium-chain fatty acids and pyruvate (Halestrap, 2012). Consequently, their expression should reflect the intestinally produced SCFA profile. Yet, there is still a lack of information to which degree the developing microbial activity in the gut influences the expression of FA receptors and transporters after birth, which awaits to be elucidated.

Besides microbial metabolites, the gut bacteria-host interactions are mediated by host recognition of conserved bacterial structures through pattern recognition receptors (**PRR**) (Metzler-Zebeli et al., 2021; Lerch et al., 2023). The expression of these receptors develops after birth (Arnaud et al., 2020; Lerch et al., 2023). Moreover, recent results emphasize the importance of the actual gut microbial composition for the expression of PRR in suckling piglets (Arnaud et al., 2020; Lerch et al., 2023). Therefore, gaining a better understanding about developmental patterns related to sensing of microbial metabolites, innate immune activation, and barrier function in the early neonatal phase is crucial to recognize developmental abnormalities. Following the development of the blood biochemical profile can also provide valid data about the general physiological health of piglets. However, there is still little information available on the developmental patterns of blood metabolites in neonatal piglets and how they associate with the developing gut.

The objective of this study was to investigate serum biochemistry and gut site-specific patterns of SCFA and expression of genes related to barrier function, innate immune response, antioxidative status and sensing of fatty and bile acids in suckling and newly weaned piglets. We hypothesized that the expression of genes related to microbial signaling would co-evolve with SCFA concentrations, whereas the expression of genes of the innate immune response would be highest on the first days of life (**DoL**) and decrease until weaning due to build-up of immune tolerance. Due to the change in diet, postweaning gene expression profiles would be different from those preweaning, with proinflammatory signaling via PRR-nuclear factor-κB elevated in the first days after weaning. Moreover, it was hypothesized that the expression of genes coding for host-related patterns involved in the microbe-host-interplay would differ from developmental patterns reported in the literature (Arnaud et al., 2020; Lerch et al., 2023) due to the strong influence of the actual gut microbiota composition.

## Materials and Methods

### Ethical statement

All procedures involving the handling and treatment of animals were approved by the institutional ethics committee of the University of Veterinary Medicine Vienna and the National authority under the Law for Animal Experiments in Austria (GZ 2020-0.437.208).

### Animals, housing, and experimental procedures

The experimental setup has been described in detail in the companion publication Yosi et al. (2024). Briefly, the experiment was conducted under practical conditions at the pig facility of the University of Veterinary Medicine Vienna (Vetfarm), consisting of two consecutive replicate batches with 10 sows (Large White) and their litters (Large White × Piétrain) each. The total number of piglets born alive was 277 and the average litter size was 13.9 ± 1.7 (SD) alive piglets at birth across both replicate batches. Handling of sows and their litters followed the standard procedures at the pig facility. The piglets were monitored from birth throughout the 28-day suckling period to 7 days after weaning. A total of 23 piglets were removed from the experiment across the 2 replicate batches due to poor health or died (either crushing or sudden death) mostly in the first week of life.

The farrowing pens (BeFree, Schauer Agrotonic GmbH, Prambachkirchen, Austria; 2.3 × 2.6 m in size) were equipped with a feeder, bowl drinker, and hayrack for the sow, and round feeder, small bowl drinker, and nest with heated flooring for the piglets. In the two replicate batches, all sows farrowed within the same 48 hours. Piglets received an iron injection on DoL4 (2 mL of Ferriphor 100 mg/mL, OGRIS Pharma Vertriebs-GmbH, Wels, Austria), followed by castration of male piglets on DoL11 (general sedation with Stresnil 40 mg/mL, 0.025 mL/kg body weight, Elanco Tiergesundheit AG, Basel, Switzerland and Narketan 100 mg/mL, 0.1 mL/kg body weight, Vetoquinol Österreich GmbH, Vienna, Austria). Piglets were vaccinated (1 mL Ingelvac CircoFLEX and 1 mL Ingelvac MycoFLEX, both from Boehringer Ingelheim GmbH, Ingelheim/Rhein, Germany) on DoL17. After 28 days, sows were separated from the farrowing pens and piglets were placed in rearing pens measuring 3.3 × 4.6 m each. Two to three litters were penned together with a maximum group size of 20 animals per pen. Nursery pens were equipped with a round feeder, two bowl drinkers, and a heated nest. Animal health was monitored daily throughout the trial and sows and piglets had free access to water throughout the entire experiment.

### Feeding

The standard feeding protocol at the pig facility was applied for suckling and weaned piglets. The composition of the milk replacer and prestarter diet can be found in Supplemental Tables S1 and S2. Piglets received creep feed that was manually prepared at least twice daily (0800 and 1500 hours) from DoL3 to 28. In the first three weeks of life, piglets got a commercial milk replacer (Supplementary Table S1). The milk replacer was prepared according to the manufacturer’s instructions and was offered in liquid form, by mixing the powder with warm water (45°C) at a ratio 1:2 [500 g/L (w/v)]. The milk replacer was fed at ambient room temperature. From DoL3 to 23 the milk replacer was offered to 100% and from DoL24 to 26 it was gradually blended with the prestarter feed (Supplementary Table S1 and S2) and provided in mash form. Blending started with a ratio of 70:30 (w/w) on DoL24, 50:50 (w/w) on DoL25, and 30:70 (w/w) on DoL26. Subsequently, the prestarter feed was fed 100% as mash on DoL27 and in dry form from DoL28 to 35. All diets used in the study met or exceeded the current recommendations for nutrient requirements (NRC, 2012).

### Blood and gut sampling

In each replicate batch and on each sampling day, 5 female and 5 male piglets with average body weight were selected for invasive sampling on DoL3, 7, 14, 21, 28, 31, and 35. In total, 10 males and 10 females were sampled on each age across the two replicate batches. One piglet was selected from each litter (with alternating sexes on the consecutive sampling days) on each sampling day. Prior to slaughter, piglets were weighed and anesthetized in the ear vein with azaperone (Stresnil 40 mg/mL, 0.025 mL/kg body weight, Azaperone, Elanco Tiergesundheit AG, Bad Homburg, Germany) and ketamine (Narketan 100 mg/mL, 0.1 mL/kg body weight, Vetoquinol Österreich GmbH, Vienna, Austria). Blood was collected from the hearts of piglets after deep sedation and placed in serum tubes (Vacuette Röhrchen CAT Serum, Greiner Bio-One International GmbH, Kremsmünster, Austria) for clinical biochemistry. The serum tubes were kept on ice until centrifugation at 3,000 × *g* for 20 min at 4°C (Eppendorf Centrifuge 5810R, Eppendorf SE, Hamburg, Germany). Following blood sampling, piglets were euthanized with embutramide via intracardiac injection (T61, 0.1 mL/kg body weight, Intervet GesmbH, Vienna, Austria). Piglets were exsanguinated by cutting the neck. Then, the abdomen was opened, and the liver and entire gut were removed aseptically. As a proxy for gut development (Pu et al., 2023), gastric and cecal lengths were measured using a measuring tape and expressed per kilogram body weight. The stomach, mid-jejunum (half of the total jejunum), cecum, and mid-colon (top of the ‘beehive’) were identified, clamped, and separated. We aimed to collect gut tissue and digesta samples from each gut site. However, the jejunum was empty in the majority of piglets. Therefore, only digesta from the stomach, cecum, and mid-colon were collected and homogenized. Digesta samples for SCFA analysis were kept on ice until storage at -20°C. For assessment of gene expression, a 10-cm-tube sample from the mid-jejunum and a 5-cm piece from the end of the cecal blind sack were taken, washed in ice-cold phosphate-buffered saline, cut into small pieces, and snap-frozen in liquid nitrogen. The serum and mucosal samples were stored at −80°C until analysis.

### Serum biochemical analysis

The analyses of serum glucose and total protein, lipids (ie, cholesterol and triglycerides), liver enzymes (ie, ALT, AST, ALP), electrolytes (ie, sodium, potassium, and chloride), and calcium and phosphate levels were conducted with enzymatic colorimetric assays using an autoanalyzer for clinical chemistry (Cobas 6000/c501; Roche Diagnostics GmbH, Rotkreuz, Switzerland) as previously described by Metzler-Zebeli et al. (2023).

### SCFA analysis

The concentrations of acetate, propionate, isobutyrate, butyrate, isovalerate, valerate, caproate, and heptanoate were analyzed using gas chromatography as described in Lerch et al. (2023). Cecal digesta amounts on DoL3 were not sufficient and therefore not analyzed for DoL3. For sample preparation, 25% ortho-phosphoric acid (4.36 mol/L), 300 μL of 4-methylvaleric acid (23.83 μmol/mL) as internal standard (Sigma-Aldrich, St Louis, MO, USA) and double-distilled water were added to 1.0 g gastric, cecal, and colonic digesta of piglets. Samples were quickly processed in an ultrasonic bath, mixed thoroughly, and centrifuged (20,000 × *g* at 4°C for 20 min). Short-chain fatty acids were measured in the clear supernatant on the GC-2010 Plus Capillary GC (Shimadzu Corp., Kyoto, Japan) using a 30 m × 0.53 mm × 0.5 μm capillary column (Trace TR Wax, Thermo Fisher Scientific, Waltham, MA, USA) and helium as carrier gas. The injection volume was 1 µL. The gas chromatograph was set up with an autosampler and injector (AOC-20s Auto Sampler; AOC-20i Auto-Injector, Shimadzu Corp., Kyoto, Japan) and a flame-ionization detector (FID-2010 Plus, Shimadzu Corp., Kyoto, Japan).

### Gene expression analyses

Total RNA from approximately 20 mg of frozen jejunal and cecal tissues was isolated with the RNeasy Mini Kit (RNeasy Mini Qiacube Kit, Qiagen, Hilden, Germany) similar to the protocol described in Lerch et al. (2023). To remove genomic DNA, the isolated RNA was treated with DNase I (Invitrogen TURBO DNA-free Kit, Thermo Fisher Scientific Inc., Waltham, MA, USA). The RNA concentration was quantified with the Qubit RNA HS Assay Kit on the Qubit 4 Fluorometer (Thermo Fisher Scientific Inc., Waltham, MA, USA). Isolates with RNA integrity numbers greater than eight were used for the synthesis of complementary DNA (**cDNA)** by use of the AB High-Capacity cDNA Reverse Transcription Kit (Thermo Fisher Scientific Inc., Waltham, MA, USA) and the Mastercycler Nexus (Eppendorf SE, Hamburg, Germany) following the manufacturer’s instructions. The cDNA was used for the amplification of 34 target genes and 5 endogenous control genes [γ-actin (**ACTG**), glyceraldehyde 3-phosphate-dehydrogenase (**GAPDH**), hypoxanthine phosphoribosyl-transferase (**HPRT**), ornithine decarboxylase antizyme 1 (**OAZ1**), and β2-microglobulin (**B2M**)]. PrimerBLAST was used to test the accuracy of the primers used for target gene and reference gene amplification (www.ncbi.nlm.nih.gov/tools/primer-blast/). Information about primers and their amplification efficiencies (E = 10^(-1/slope)^) are provided in Supplementary Table S3. A robot (epMotion 5075 TMX, Eppendorf SE, Hamburg, Germany) was used for qPCR pipetting. The innuMIX qPCR DS Green Standard (IST Innuscreen, Berlin, Germany) was used for amplification and quantification of cDNA in duplicates on the qTower384 (Analytik Jena, Jena, Germany). Each standard and sample reaction (10 µl) comprising 7 µl master mix, forward and reverse primers (200 nM each), and 3 µl (25 ng) cDNA template were included. Furthermore, 40 cycles of 95°C for 30 s followed by primer annealing and elongation at 60°C for 60 s were performed after a 2-min initial denaturation step at 95°C. At each step, the fluorescence was measured. Melting curves were created to test the specificity of PCR amplification. Negative template controls, as well as serial dilutions of the standards, were run in triplicates. To normalize raw gene expression and determine ΔCt values for relative gene expression levels, the geometric mean of the most stably expressed reference genes (*B2M* and *HPRT*) was used. The 2^-ΔΔCt^ method was used to calculate relative gene expression. The ΔΔCt values were calculated using the sample with the highest expression (lowest ΔCt) of the respective target gene.

### Statistical analysis

The Shapiro–Wilk test with the UNIVARIATE procedure in SAS (version 9.4; SAS Inst. Inc., Cary, NC) was used to test the normal distribution of the residuals of all variables. The data were transformed using the Boxcox method and the Transreg procedure in SAS if the residuals were not normally distributed. All data from piglets, i.e., gut size, SCFA, host mucosal gene expression, and serum biochemical parameters, were subjected to ANOVA using the MIXED procedure in SAS. A random model was used to investigate the fixed effect of increasing age (ie, DoL) of piglets on parameters. Sex was first included in the initial model as covariate but excluded from the final model due to very few sex effects. The random effect was animal. Day of life nested within replicate batch and litter represented the experimental unit. Degrees of freedom were approximated by the Kenward-Rogers method (ddfm = kr). The Bonferroni post hoc text was applied to perform multiple pairwise comparisons among least-square means. Data were reported as the least-square means ± standard errors of the means (**SEM**). A significant difference was defined at *P *≤ 0.05 and trends at 0.05 < *P* ≤ 0.10. Pearson correlation coefficients were calculated using PROC CORR in SAS to identify associations between 1) gastric and cecal size and their respective SCFA concentrations, and 2) gene expression at cecal mucosa and SCFA concentrations in cecal digesta, separately for the preweaning and postweaning period. If *P *< 0.05, only correlations with r > 0.30 were considered relevant. To visualize the obtained correlations, heat maps were generated using the levelplot() function in the lattice package in R Studio (version 2023.06.0).

## Results

Due to the removal of piglets on sampling days and of those that were medically treated or died, the average number of suckling piglets per litter decreased from birth to weaning and was 12.2, 10.9, 9.8 and 8.8 ± 2.1 (SD) on DoL6, 13, 20 and 27 of life, respectively. The amount of creep feed consumed daily by piglets was relatively similar throughout the suckling period and amounted to 20 g/day and piglet (Supplementary Table S4; Yosi et al., 2024).

### Changes in serum biochemical profile

The concentration of glucose in serum increased from DoL3 to 28 but decreased back to the level of DoL7 after weaning (*P *< 0.001; Table 1). The total protein was relatively stable during the suckling phase and only lower on DoL35 compared to DoL3 (*P < *0.05). Triglycerides fluctuated between 96 and 134 mg/dL between DoL3 to 28 but decreased after weaning to 49 and 63 mg/dL on DoL 31 and 35, respectively (*P < *0.001). Cholesterol levels rose from 85 mg/dL on DoL3 to 135 mg/dL by DoL14, then gradually decreased to 67 mg/dL until DoL35 (*P < *0.001). The liver enzyme ALP steadily decreased from DoL7 to 21 and remained stable thereafter (*P < *0.001). The activities of the liver enzymes AST and ALT were greatest on DoL3 and decreased to DoL7, after which the activities remained stable (*P < *0.001). The serum calcium, phosphate and potassium levels were higher either on DoL3, 7 or 14 compared to the other DoL (*P < *0.001). Serum levels of sodium and chloride were higher on DoL3, 31 (only chloride) and 35 compared to the other DoL (*P < *0.001).

**Table 1.** Age-related development of biochemical profiles in serum of suckling and newly weaned piglets

Day of life	3	7	14	21	28	31	35	SEM	*P*-value
Glucose, mg/dL	106^c^	134^b^	148^ab^	149^ab^	155^a^	139^bc^	131^b^	4.10	<0.001
Total protein, g/dL	5.16^a^	5.07^ab^	4.75^ab^	4.88^ab^	4.69^ab^	4.82^ab^	4.63^b^	0.11	0.012
Triglycerides, mg/dL	115^ab^	134^a^	95.5^ab^	102^ab^	117^a^	49.4^c^	63.4^bc^	12.31	<0.001
Cholesterol, mg/dL	85.1^bc^	124^ab^	135^a^	123^ab^	116^b^	101^b^	66.5^c^	6.15	<0.001
Alkaline phosphatase, U/L	1959^a^	1636^a^	1008^b^	495^bc^	387^c^	317^c^	280^c^	126.9	<0.001
Aspartate transaminase, U/L	112^a^	48.1^b^	54.3^b^	42.4^b^	33.1^b^	25.8^b^	48.9^b^	10.05	<0.001
Alanine transaminase, U/L	80.3^a^	30.7^b^	30.1^b^	32.0^b^	35.0^b^	40.7^b^	45.9^b^	4.02	<0.001
Calcium, mmol/L	2.65^b^	2.91^a^	2.73^b^	2.69^b^	2.69^b^	2.47^c^	2.54^c^	0.03	<0.001
Phosphate, mmol/L	2.44^c^	2.96^a^	3.17^a^	2.83^b^	2.81^b^	2.42^c^	2.50^bc^	0.08	<0.001
Sodium, mmol/L	143^a^	138^bc^	136^bc^	134^c^	134^c^	138^ab^	140^a^	1.02	<0.001
Potassium, mmol/L	6.00^a^	5.04^b^	4.87^b^	4.65^b^	4.54^b^	4.70^b^	4.90^b^	0.19	<0.001
Chloride, mmol/L	105^a^	102^ab^	100^bc^	98.2^bc^	97.8^c^	105^a^	107^a^	0.98	<0.001

Values are presented as least squares means ± SEM. Piglets were weaned on day 28 of life. At each time point, 20 piglets (10 male and 10 female piglets) were sampled.

a,b,c,d Means without a common superscript in the same row differ (*P *< 0.05).

### Development of stomach and cecum size

The size of stomach per kilogram body weight was higher on DoL3 and 7 compared to DoL21, 28 and 31 (*P *< 0.05, Table 2). Day of life similarly affected the size of the cecum (*P *= 0.042). Stomach and cecum size did not correlate with luminal SCFA concentrations in the suckling and early postweaning period (data not shown).

**Table 2.** Age-related development of stomach and cecum size (cm/kg body weight) in suckling and newly weaned piglets

Day of life	3	7	14	21	28	31	35	SEM	*P*-value
Stomach	3.93^a^	3.14^a^	2.26^ab^	1.65^b^	1.42^b^	1.46^b^	2.37^ab^	0.265	<0.001
Cecum	1.51	1.58	1.35	1.11	1.00	1.09	1.57	0.165	<0.042

Values are presented as least squares means ± SEM. Weaning took place on day 28 of life. At each time point, 20 piglets (10 male and 10 female piglets) were sampled.

a,b,c,d Means without a common superscript in the same row differ (*P *< 0.05).

### Age-related changes in SCFA concentrations in gastric, cecal, and colonic digesta

Age influenced the concentration of total SCFA in gastric digesta (*P *< 0.05; Table 3). The greatest change was the decrease in SCFA from DoL28 to 31 (*P *< 0.05), which was mainly due to changes in acetate and isobutyrate. By contrast, the concentration of caproate was stable during the suckling period but increased after weaning (*P *< 0.05). In cecal digesta, total and individual SCFA were affected by age (*P *< 0.05) and followed the same pattern in that they increased until weaning on DoL28. The concentration of acetate, isobutyrate and isovalerate (DoL31), and total SCFA (DoL35) in cecal digesta dropped after weaning, whereas the concentration of butyrate increased from DoL21 to 35 (*P *< 0.05). Regarding the colon, the concentration of acetate, propionate, isobutyrate, butyrate, valerate, and total SCFA increased from DoL3 to 35 (*P *< 0.05). In contrast, the concentration of isovalerate increased until DoL21 and then decreased to DoL35 (*P *< 0.05).

**Table 3.** Age-related development of short-chain fatty acid (SCFA) concentrations in the gastric, cecal, and colonic digesta of suckling and newly weaned piglets

Day of life	3	7	14	21	28	31	35	SEM	*P*-value
Stomach, µmol/g digesta									
Total SCFA	23.5^ab^	12.9^ab^	14.4^ab^	12.6^ab^	24.0^a^	8.94^b^	19.9^ab^	3.325	0.007
Acetate	20.1	11.7	13.0	10.2	20.5	7.64	18.3	3.050	0.015
Propionate	1.96	0.98	1.01	1.82	1.88	0.75	0.82	0.446	0.213
Isobutyrate	0.110	0.011	0.013	0.014	0.074	0.008	0	0.025	0.028
Butyrate	1.02	0.14	0.20	0.35	0.73	0.056	0.10	0.292	0.132
Caproate	0.080^c^	0.048^c^	0.058^c^	0.070^c^	0.13^bc^	0.46^ab^	0.67^a^	0.078	<0.001
Cecum, µmol/g digesta									
Total SCFA	.	43.9^c^	105^b^	132^ab^	152^a^	124^ab^	114^b^	8.775	<0.001
Acetate	.	28.1^c^	63.8^b^	79.3^ab^	92.0^a^	68.4^b^	61.4^b^	5.056	<0.001
Propionate	.	9.4^c^	22.9^b^	29.1^ab^	31.9^ab^	34.3^a^	30.0^ab^	2.239	<0.001
Isobutyrate	.	1.04^b^	3.19^a^	3.84^a^	4.35^a^	1.38^b^	0.67^b^	0.331	<0.001
Butyrate	.	2.82^d^	8.25^cd^	10.4^bc^	13.7^ab^	14.5^ab^	16.8^a^	1.267	<0.001
Isovalerate	.	1.21^b^	3.40^a^	4.02^a^	4.21^a^	1.13^b^	0.63^b^	0.316	<0.001
Valerate	.	1.13^b^	3.40^ab^	4.38^a^	5.18^a^	3.83^a^	4.27^a^	0.486	<0.001
Caproate	.	0.14^b^	0.35^ab^	0.49^ab^	0.66^a^	0.61^a^	0.49^ab^	0.094	0.008
Heptanoate	.	0.24	0.18	0.23	0.55	0.45	0.34	0.114	0.032
Colon, µmol/g digesta									
Total SCFA	33.0^b^	44.7^b^	78.1^b^	94.3^b^	82.6^b^	135^a^	158^a^	13.649	<0.001
Acetate	21.0^c^	28.8^bc^	48.2^bc^	57.3^bc^	52.8^bc^	79.5^ab^	92.0^a^	8.246	<0.001
Propionate	6.93^b^	9.56^b^	17.3^b^	21.9^ab^	17.3^b^	34.0^a^	37.4^a^	3.291	<0.001
Isobutyrate	0.74^b^	1.05^b^	2.12^ab^	2.46^a^	2.05^ab^	2.09^ab^	1.97^a^	0.341	0.019
Butyrate	2.50^c^	2.91^c^	5.71^c^	6.73^c^	5.93^c^	13.19^b^	19.56^a^	1.588	<0.001
Isovalerate	0.93^b^	1.02^b^	2.04^ab^	2.63^a^	2.21^a^	2.16^ab^	2.03^b^	0.278	<0.001
Valerate	0.73^b^	0.93^b^	2.07^b^	2.44^b^	1.83^b^	2.87^b^	4.53^a^	0.419	<0.001
Caproate	0.07	0.34	0.68	0.69	0.44	0.68	0.60	0.115	0.148
Heptanoate	0.03	0.20	0.38	0.22	0.03	0.22	0.23	0.096	0.143

Values are presented as least squares means ± SEM. Mainly acetate, proprionate, isobutyrate, butyrate, and caproate were detected in gastric digesta. Piglets were weaned on day 28 of life. The amount of cecal digesta on day 3 of life was not sufficient to allow measuring SCFA concentrations. At each time point, 20 piglets (10 male and 10 female piglets) were sampled.

a,b,c,d,e Means without a common superscript in the same row differ (*P *< 0.05).

### Age-related changes in gene expression at the jejunal mucosa

Jejunal expression of *FFAR1*, *HCAR1*, *MCT1*, and farnesoid X receptor (**FXR***)* was higher on DoL3 and/or 7 but decreased as the piglets got older, whereby the expression of *MCT1* increased again from DoL31 to 35 (*P *< 0.05, Table 4). The opposite was true for sodium-coupled monocarboxylate transporter-1 (**SMCT1**) expression, which gradually increased until it peaked on DoL35 (*P *< 0.001). The expression of *FFAR4* and *SMCT2* decreased during the suckling phase, but it increased to peak on DoL35 (*P *< 0.05). The expression of *FFAR2* increased from DoL3 to 21 and decreased again after weaning, whereas *FFAR3* was highest expressed on DoL7 and decreased thereafter (*P *< 0.05).

**Table 4.** Age-related development of relative expression of genes in the jejunum of suckling and newly weaned piglets

Day of life	3	7	14	21	28	31	35	SEM	*P*-value
Short-chain fatty acid receptors and transporters									
*FFAR1*	0.38^ab^	0.44^a^	0.29^b^	0.14^c^	0.17^bc^	0.13^c^	0.13^c^	0.029	<0.001
*FFAR2*	0.08^b^	0.12^b^	0.12^ab^	0.12^a^	0.14^a^	0.07^b^	0.08^b^	0.011	<0.001
*FFAR3*	0.54^ab^	0.66^a^	0.42^b^	0.23^c^	0.18^c^	0.14^c^	0.11^c^	0.034	<0.001
*FFAR4*	0.025^b^	0.028^ab^	0.027^ab^	0.021^b^	0.028^b^	0.023^b^	0.037^a^	0.002	<0.001
*HCAR1*	0.064^a^	0.068^a^	0.057^ab^	0.044^ab^	0.042^ab^	0.036^b^	0.032^b^	0.006	<0.001
*MCT1*	0.077^a^	0.058^b^	0.035^c^	0.036^c^	0.039^c^	0.021^d^	0.034^c^	0.003	<0.001
*SMCT1*	0.22^c^	0.30^bc^	0.41^b^	0.44^ab^	0.58^a^	0.36^bc^	0.57^a^	0.035	<0.001
*SMCT2*	0.20^b^	0.19^b^	0.18^bc^	0.09^c^	0.08^c^	0.27^ab^	0.34^a^	0.031	<0.001
Bile acid receptor									
*FXR*	0.18^a^	0.14^b^	0.09^c^	0.11^bc^	0.10^bc^	0.06^c^	0.09^bc^	0.011	<0.001
Pattern-recognition receptors									
*TLR1*	0.23^a^	0.23^a^	0.19^ab^	0.14^bc^	0.16^bc^	0.10^c^	0.14^bc^	0.015	<0.001
*TLR2*	0.031^bc^	0.059^a^	0.028^bc^	0.029^bc^	0.036^b^	0.016^c^	0.014^c^	0.005	<0.001
*TLR4*	0.065^bc^	0.074^b^	0.082^ab^	0.10^ab^	0.13^a^	0.057^c^	0.073^bc^	0.008	<0.001
*TLR5*	0.23	0.32	0.32	0.31	0.33	0.21	0.24	0.028	0.005
*TLR6*	0.19^ab^	0.23^a^	0.23^a^	0.18^ab^	0.21^ab^	0.14^b^	0.20^ab^	0.017	0.004
*TLR7*	0.18^c^	0.23^bc^	0.28^b^	0.35^ab^	0.44^a^	0.27^bc^	0.33^ab^	0.026	<0.001
*TLR8*	0.076^b^	0.093^b^	0.15^b^	0.27^a^	0.36^a^	0.14^b^	0.15^b^	0.022	<0.001
*TLR9*	0.24^bc^	0.28^bc^	0.33^b^	0.34^ab^	0.42^a^	0.18^c^	0.19^c^	0.029	<0.001
*NOD1*	0.078^a^	0.086^a^	0.077^a^	0.074^ab^	0.088^a^	0.043^bc^	0.034^c^	0.008	<0.001
*NOD2*	0.16^b^	0.18^ab^	0.17^ab^	0.20^ab^	0.25^a^	0.10^b^	0.11^b^	0.020	<0.001
Transcription factor and cytokines									
*NKAP*	0.10^ab^	0.12^a^	0.097^ab^	0.063^ab^	0.064^ab^	0.067^ab^	0.057^b^	0.013	0.004
*TNFA*	0.078^c^	0.13^b^	0.13^b^	0.15^ab^	0.17^a^	0.15^ab^	0.10^bc^	0.013	<0.001
*IL1B*	0.011	0.015	0.019	0.030	0.033	0.032	0.032	0.006	0.060
*IL6*	0.048	0.067	0.068	0.088	0.079	0.069	0.038^c^	0.012	0.075
*IL10*	0.14^c^	0.23^bc^	0.24^b^	0.34^a^	0.38^a^	0.23^bc^	0.21^bc^	0.022	<0.001
*TGFB1*	0.18^c^	0.25^bc^	0.28^bc^	0.32^b^	0.43^a^	0.29^bc^	0.28^bc^	0.029	<0.001
Antioxidative enzymes									
*SOD1*	0.28^b^	0.34^a^	0.23^bc^	0.31^ab^	0.28^b^	0.16^c^	0.28^b^	0.021	<0.001
*GPX1*	0.31^a^	0.32^a^	0.16^b^	0.16^b^	0.20^b^	0.14^b^	0.18^b^	0.024	<0.001
Barrier function genes									
*MUC2*	0.085^ab^	0.079^b^	0.085^ab^	0.076^b^	0.11^a^	0.090^ab^	0.12^a^	0.008	<0.001
*MUC4*	0.019^a^	0.029^a^	0.020^a^	0.010^b^	0.014^ab^	0.007^b^	0.007^b^	0.002	<0.001
*CLDN1*	0.025	0.031	0.034	0.028	0.029	0.029	0.015	0.006	0.457
*CLDN4*	0.18^b^	0.28^ab^	0.33^a^	0.36^a^	0.32^a^	0.19^b^	0.24^ab^	0.023	<0.001
*OCLN*	0.36^b^	0.55^a^	0.43^ab^	0.35^b^	0.46^ab^	0.32^b^	0.46^ab^	0.037	0.003
*ZO1*	0.39^a^	0.38^a^	0.27^b^	0.20^bc^	0.25^bc^	0.17^c^	0.24^bc^	0.021	<0.001
*IAP*	0.29^a^	0.28^a^	0.12^b^	0.071^b^	0.075^b^	0.061^c^	0.072^b^	0.025	<0.001

Values are presented as least squares means ± standard error of the mean (SEM). Piglets were weaned on day 28 of life. At each time point, 10 piglets (5 male and 5 female piglets) were sampled. *FFAR1,-2,-3,-4*, free fatty acid receptor 1,-2,-3,-4; *HCAR1*, hydroxycarboxylic acid receptor 1; *MCT1*, monocarboxylate transporter 1; *SMCT1,-2*, sodium-coupled monocarboxylate transporter 1,-2; *FXR*, farnesoid X receptor; *TLR1,-2,-4,-5,-6,-7,-8,-9*, Toll-like receptor 1,-2,-4,-5,-6,-7,-8,-9; *NOD1,-2*, nucleotide-binding oligomerization domain 1,-2; *NKAP*, NFKB activating protein; *TNFA*, tumor necrosis factor alpha; *IL1B,-6,-10*, interleukin 1 beta,-6,-10; *TGFB1*, transforming growth factor beta 1; *SOD1*, superoxide dismutase 1; *GPX1*, glutathione peroxidase 1; *MUC2,-4*, mucin 2,-4; *CLDN1,-4*, claudin 1,-4; *OCLN*, occludin; *ZO1*, zonula occludens-1; *IAP*, intestinal alkaline phosphatase.

a,b,c,d,e Means without a common superscript in the same row differ (*P *< 0.05).

The expression of toll-like receptor (**TLR)**-1 and *TLR2* decreased from DoL7 to 31 (*P *< 0.05). By contrast, expression of *TLR4*, *TLR7*, *TLR8*, *TLR9*, and nucleotide-binding oligomerization domain (**NOD)-**2 increased until weaning and dropped postweaning (*P *< 0.05). Moreover, the expression of *TLR6* was higher during the suckling period but decreased on DoL31 (*P *< 0.05). Jejunal *NOD1* was similarly expressed before weaning and dropped thereafter (*P *< 0.05). Jejunal expression of nuclear factor kappa B-activating protein (**NKAP**) was highest on DoL7 and gradually decreased afterwards (*P *< 0.05). Gene expression of proinflammatory tumor necrosis factor-alpha (**TNFA)** had its maximum on DoL28 and decreased on DoL35 (*P *< 0.05). Expression of *IL10* and transforming growth factor beta (**TGFB)**-1 increased preweaning until DoL28 and dropped postweaning (*P *< 0.001). Genes coding for antioxidative enzymes were differently expressed. Expression of superoxide dismutase (**SOD)-**1 fluctuated throughout the suckling and postweaning period (*P *< 0.05), whereas glutathione peroxidase (**GPX**)-1 was higher expressed on DoL3 and 7 compared to the other DoL (*P *< 0.05). The expression of all genes coding for mucins and tight-junction proteins were affected by age (*P *< 0.001) except claudin (**CLDN**)-1 (*P *> 0.05). Expression of mucin (**MUC**)-2 and *CLDN4* increased until DoL28, then dropped on DoL31 and recovered on DoL35 (*P *< 0.001). Regarding zonula occludens (**ZO**)-1, intestinal alkaline phosphatase (**IAP)** and *MUC4*, their expression was highest on DoL3 and/or 7, respectively, and then decreased (*P *< 0.001). Occludin (**OCLN**) was highest expressed on DoL7 and fluctuated on the following DoL (*P *< 0.001).

**Figure 1.**
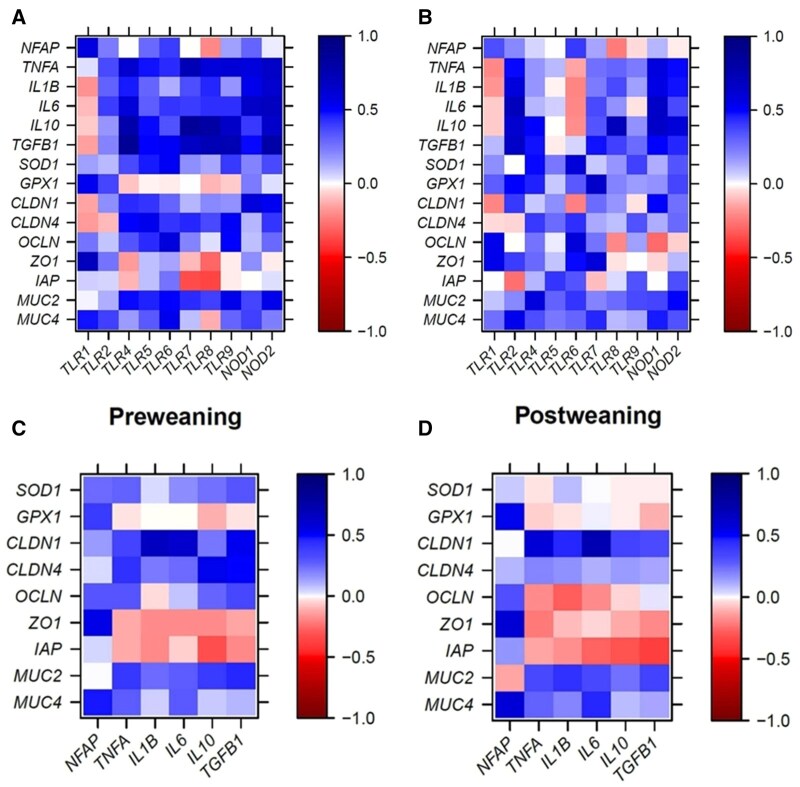
Pearson correlation heatmaps for the jejunum showing associations of expression levels of PRR with those of cytokines and barrier function genes for the suckling (A) and early postweaning period (B), as well as associations between expression levels of cytokines with those of barrier function genes for the suckling (C) and early postweaning period (D).

Correlations between expression levels of genes within the PRR-cytokine signaling pathways in the jejunum showed that preweaning the expression of PRR positively correlated with the transcription factor *NKAP* (*r *> 0.30; *P *< 0.05; Figure 1A). Many positive correlations were also found between expression levels of PRR and expression levels of pro- and anti-inflammatory cytokines, *SOD1*, and *GPX1* in both pre- and postweaning period (*P *< 0.05; Figure 1A and B). Likewise, the expression of *TLR1 to TLR9*, *NOD1*, and *NOD2* was positively correlated with the expression levels of many but not all genes coding for cytokines and tight-junction proteins preweaning (*r *> 0.20; *P *< 0.05), whereas the expression of *TLR7* and *TLR8* negatively correlated with the expression of *IAP* and *ZO1* preweaning (*r* > -0.36; *P *< 0.05). In addition, jejunal *NKAP* expression was positively correlated with expression levels of *OCLN*, *ZO1*, *MUC4*, *SOD1*, and *GPX1* in the preweaning period (*r *> 0.25; *P *< 0.05; Figure 1C). Positive correlations were also found between the expression levels of *TNFA*, *IL1B*, *IL6*, *IL10*, and *TGFB1* with *CLDN1*, *CLDN4*, *OCLN*, *MUC2*, *MUC4*, or *SOD1* more for the preweaning period, less for the postweaning period (*P *< 0.05; Figure 1C and D).

### Age-related changes in gene expression at the cecal mucosa

At the cecal mucosa, the expression of *FFAR2*, *FFAR3*, and *SMCT2* was highest on DoL3 and decreased afterwards (*P *< 0.05; Table 5). Similarly, *HCAR1* was equally expressed during the suckling period, but decreased after weaning (*P *< 0.001). In contrast, the expression of *FFAR4* was lowest on DoL7, but then gradually increased to its maximum expression on DoL35 (*P *< 0.001). The expression of the bile acid receptor *FXR* was highest on DoL3 and gradually decreased afterwards (*P *< 0.05). The expression of the PRR *TLR1*, *TLR2*, *TLR5*, *TLR6*, *NOD1*, and *NOD2* were highest on DoL3 and that of *TLR9* was highest on DoL7 and subsequently decreased (*P *< 0.05). In contrast, the expression of *TLR4* and *TLR8* increased from DoL3 to 35, respectively (*P *< 0.05). The expression of *TLR7* increased from DoL7 until DoL28 (*P *< 0.05). The expression of *NKAP* and *TGFB1* decreased after DoL3 (*P *< 0.05). In contrast, the expression of *IL10* was lowest on DoL3 and 7 but increased toward DoL35 (*P *< 0.001). *TNFA*, *IL1B* and *IL6* were highest expressed at DoL3, decreased thereafter, and increased again toward DoL35 (*P *< 0.05). Cecal expression of *SOD1* decreased throughout the suckling period and remained more or less stable after weaning (*P *< 0.05). Expression of *MUC2* decreased from DoL3 to 7, increased to DoL28 but decreased again postweaning (*P *< 0.001). Expression of *CLDN1*, *ZO1*, and *IAP* decreased from DoL3 to 35 (*P *< 0.001), whereas *CLDN4* and *OCLN* were highest expressed on DoL7 and decreased thereafter (*P *< 0.001).

**Table 5.** Age-related development of relative expression of genes in the cecum of suckling and newly weaned piglets

Day of life	3	7	14	21	28	31	35	SEM	*P*-value
Short-chain fatty acid receptors and transporters									
*FFAR1*	0.23	0.18	0.22	0.20	0.20	0.11	0.12	0.033	0.068
*FFAR2*	0.33^a^	0.16^b^	0.14^b^	0.17^b^	0.18^b^	0.18^b^	0.20^b^	0.026	<0.001
*FFAR3*	0.31^a^	0.21^b^	0.14^c^	0.14^c^	0.14^c^	0.06^d^	0.05^d^	0.012	<0.001
*FFAR4*	0.42^ab^	0.32^b^	0.41^ab^	0.43^ab^	0.40^ab^	0.50^a^	0.54^a^	0.037	0.002
*HCAR1*	0.37^a^	0.36^a^	0.34^a^	0.34^a^	0.38^a^	0.17^b^	0.12^b^	0.032	<0.001
*MCT1*	0.32	0.31	0.38	0.39	0.41	0.34	0.36	0.034	0.325
*SMCT1*	0.007	0.010	0.009	0.002	0.004	0.003	0.005	0.003	0.525
*SMCT2*	0.18^a^	0.012^b^	0.012^b^	0.001^b^	0.001^b^	0.0001^b^	0.0001^b^	0.017	<0.001
Bile acid receptor									
*FXR*	0.53^a^	0.19^b^	0.13^bc^	0.078^bc^	0.082^bc^	0.060^c^	0.026^c^	0.025	<0.001
Pattern-recognition receptors									
*TLR1*	0.62^a^	0.47^ab^	0.44^b^	0.48^ab^	0.53^ab^	0.33^b^	0.41^b^	0.038	<0.001
*TLR2*	0.53^a^	0.35^b^	0.30^bc^	0.35^b^	0.34^b^	0.21^c^	0.27^bc^	0.028	<0.001
*TLR4*	0.23^b^	0.24^ab^	0.23^b^	0.25^ab^	0.29^ab^	0.28^ab^	0.34^a^	0.023	0.008
*TLR5*	0.62^a^	0.44^b^	0.37^b^	0.35^bc^	0.39^b^	0.23^c^	0.22^c^	0.031	<0.001
*TLR6*	0.67^a^	0.60^a^	0.52^ab^	0.53^a^	0.60^a^	0.31^c^	0.37^bc^	0.037	<0.001
*TLR7*	0.38^ab^	0.31^b^	0.38^ab^	0.45^ab^	0.49^a^	0.39^ab^	0.44^ab^	0.034	0.023
*TLR8*	0.16^c^	0.17^c^	0.24^bc^	0.33^ab^	0.37^ab^	0.34^ab^	0.40^a^	0.031	<0.001
*TLR9*	0.31^ab^	0.43^a^	0.39^a^	0.37^a^	0.35^ab^	0.24^b^	0.29^ab^	0.028	<0.001
*NOD1*	0.52^a^	0.35^b^	0.29^b^	0.33^b^	0.32^b^	0.26^b^	0.24^b^	0.032	<0.001
*NOD2*	0.45^a^	0.36^ab^	0.32^b^	0.35^ab^	0.38^ab^	0.38^ab^	0.36^ab^	0.027	0.046
Transcription factor and cytokines									
*NKAP*	0.34^a^	0.22^b^	0.14^b^	0.13^b^	0.16^b^	0.11^b^	0.10^b^	0.030	<0.001
*TNFA*	0.28^a^	0.17^b^	0.14^b^	0.16^b^	0.17^b^	0.23^ab^	0.24^a^	0.022	<0.001
*IL1B*	0.23^a^	0.038^c^	0.045^c^	0.081^bc^	0.078^bc^	0.18^ab^	0.14^ab^	0.031	<0.001
*IL6*	0.30^a^	0.17^b^	0.11^b^	0.10^b^	0.10^b^	0.17^b^	0.12^b^	0.024	<0.001
*IL10*	0.19^c^	0.18^c^	0.24^c^	0.30^bc^	0.35^b^	0.35^b^	0.45^a^	0.029	<0.001
*TGFB1*	0.57^a^	0.54^a^	0.37^b^	0.35^b^	0.40^b^	0.38^b^	0.40^b^	0.027	<0.001
Antioxidative enzymes									
*SOD1*	0.49^a^	0.40^a^	0.37^b^	0.32^b^	0.26^c^	0.33^bc^	0.29^bc^	0.030	<0.001
*GPX1*	0.34	0.29	0.26	0.27	0.29	0.32	0.32	0.026	0.319
Barrier function genes									
*MUC2*	0.33^a^	0.17^b^	0.23^ab^	0.29^ab^	0.31^a^	0.22^ab^	0.16^c^	0.029	<0.001
*MUC4*	0.40^ab^	0.40^ab^	0.33^ab^	0.37^ab^	0.37^ab^	0.47^a^	0.28^b^	0.041	0.058
*CLDN1*	0.58^a^	0.48^a^	0.28^b^	0.22^bc^	0.24^bc^	0.19^bc^	0.14^c^	0.031	<0.001
*CLDN4*	0.28^c^	0.47^a^	0.31^bc^	0.22^bc^	0.25^bc^	0.35^ab^	0.19^c^	0.031	<0.001
*OCLN*	0.49^b^	0.58^a^	0.44^ab^	0.37^bc^	0.39^bc^	0.28^c^	0.27^c^	0.037	<0.001
*ZO1*	0.69^a^	0.54^ab^	0.55^ab^	0.48^b^	0.50^b^	0.26^c^	0.23^c^	0.033	<0.001
*IAP*	0.014^a^	0.0007^b^	0.0005^b^	0.0002^b^	0.0001^b^	0.00003^b^	0.0002^b^	0.002	<0.001

Values are presented as least squares means ± SEM. Piglets were weaned on day 28 of life. At each time point, 10 piglets (5 male and 5 female piglets) were sampled. *FFAR1,-2,-3,-4*, free fatty acid receptor 1,-2,-3,-4; *HCAR1*, hydroxycarboxylic acid receptor 1; *MCT1*, monocarboxylate transporter 1; *SMCT1,-2*, sodium-coupled monocarboxylate transporter 1,-2; *FXR*, farnesoid X receptor; *TLR1,-2,-4,-5,-6,-7,-8,-9*, Toll-like receptor 1,-2,-4,-5,-6,-7,-8,-9; *NOD1,-2*, nucleotide-binding oligomerization domain 1,-2; *NKAP*, NFKB activating protein; *TNFA*, tumor necrosis factor alpha; *IL1B,-6,-10*, interleukin 1 beta,-6,-10; *TGFB1*, transforming growth factor beta 1; *SOD1*, superoxide dismutase 1; *GPX1*, glutathione peroxidase 1; *MUC2,-4*, mucin 2,-4; *CLDN1,-4*, claudin 1,-4; *OCLN*, occludin; *ZO1*, zonula occludens-1; *IAP*, intestinal alkaline phosphatase.

a,b,c,d,e Means without a common superscript in the same row differ (*P *< 0.05).

**Figure 2.**
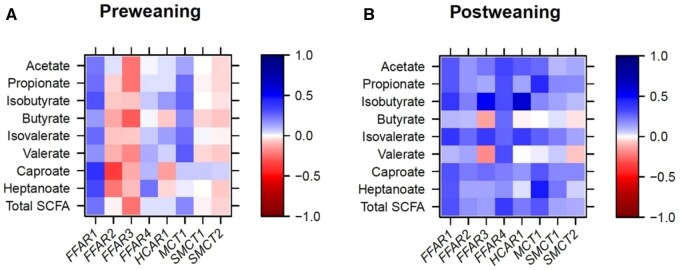
Pearson correlation heatmap showing associations between short-chain fatty acid concentrations in cecal digesta and expression of fatty acid receptors and transporters at the cecal mucosa for the suckling (A) and early postweaning period (B).

**Figure 3.**
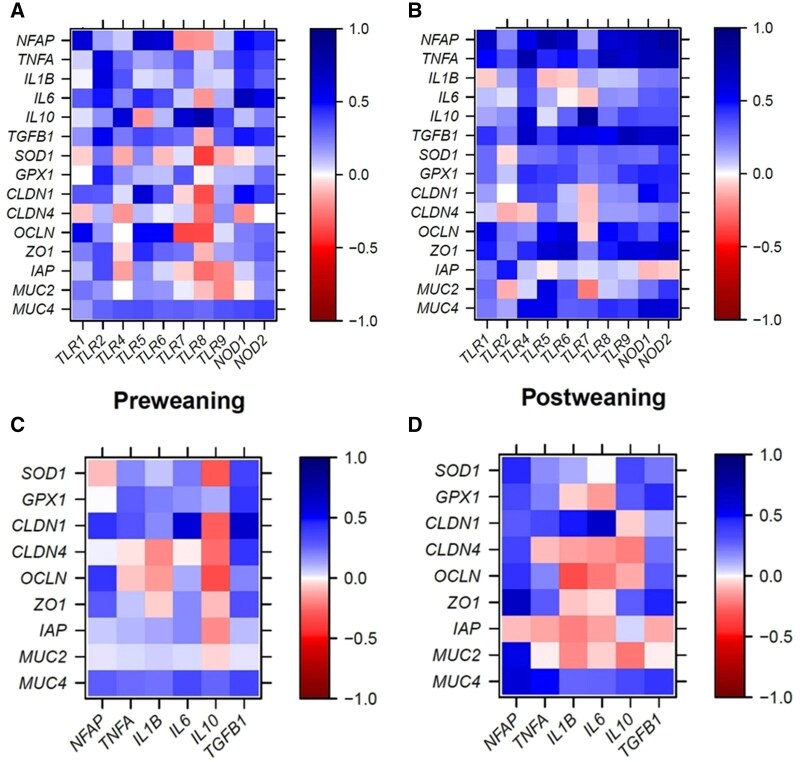
Pearson correlation heatmaps for the cecum showing associations of expression levels of PRR with those of cytokines and barrier function genes for the suckling (A) and early postweaning period (B), as well as associations between expression levels of cytokines with those of barrier function genes for the suckling (C) and early postweaning period (D).

Positive correlations existed between cecal *FFAR1* expression and concentrations of isobutyrate, caproate, and heptanoate during the suckling phase (*r *> 0.30; *P *< 0.05; Figure 2A), whereas the expression of *FFAR2* correlated negatively with caproate (*r *= -0.36; *P *< 0.05). Postweaning, cecal expression of *FFAR1*, *FFAR3*, and *HCAR1* was positively correlated with isobutyrate, and isovalerate (*r *> 0.37; *P *< 0.05; Figure 2B). Positive correlations were also found between *FFAR4* expression and acetate, propionate, isobutyrate, and total SCFA (*r *> 0.32; *P *< 0.05) as well as between expression of *MCT1* and propionate and heptanoate postweaning (*r *> 0.44; *P *< 0.05). Like in the jejunum, PRR expression correlated to *NKAP* and cytokine expression in the cecum but for both pre- and postweaning period (*r *> 0.30; *P *< 0.05; Figure 3A and B). Less positive correlations were found in the cecum compared to the jejunum between expression levels of PRR and expression levels of pro- and anti-inflammatory cytokines, tight-junction protein and mucin genes pre- and postweaning (*r *> 0.30; *P *< 0.05). There were negative relationship of *TLR8* expression with *SOD1*, *CLDN1*, and *OCLN* expression levels preweaning. Expression of *NKAP* was positively correlated to *CLDN1* and *OCLN* (*r *> 0.42; *P *< 0.05; Figure 3C). There were further positive correlations between *TGFB1* and many barrier function genes preweaning but less postweaning (*r *> 0.34; *P *< 0.05; Figure 3C and 3D).

## Discussion

As reference values for neonatal piglets are still few, the present study adds referable data for clinical diagnostics and gut development in healthy animals to existing knowledge (e.g., Lerch et al., 2023; Zheng et al., 2024). Present results demonstrate differential expression patterns of genes related to SCFA and bile acid sensing, PRR, and barrier function in the jejunum and cecum from birth to weaning and thereafter. Compared to previous research (Arnaud et al., 2020; Tang et al., 2022; Lerch et al., 2023), the age-related gene expression patterns were partially specific to this study, indicating that comparison of age-related expression patterns of genes between studies can be helpful to disentangle the influence of the microbial presence on gene transcription in the first weeks of life. Detailed information on the age-related development of the gastric and cecal bacterial and fungal microbiome in the piglets can be found in Yosi et al. (2024). Overall, it is important to note that mRNA may not always correspond to the amount of functional protein (Steele et al. 2012) but it is indicative for the induction of a molecular response due to a luminal stimulus (Vötterl et al., 2023).

The serum activities of AST and ALT are considered indicators of soft tissue damage including altered membrane permeability (Nyblom et al. 2004). Their presently observed high activities on DoL3 compared to DoL7 or 14 likely displayed the immaturity of the liver and intestine at this age (Wang et al. 2021). The fact that piglets were not fasted before blood sampling may partly explain the rising serum glucose levels during the suckling period; an observation that contrasted with previous observations (Metzler-Zebeli et al., 2023). They could be due to the increasing milk intake and, to a lesser extent, the intake of creep feed by piglets with increasing age. Piglets change from glucogenic to ketogenic metabolism after birth (Mellor and Cockburn, 1986). The question arises whether a daily intake of 20 g (dry matter basis) of starchy creep feed was sufficient to shift the metabolism of the piglet back to favor glucose instead of fat as energy substrate. In addition to the low creep feed, it can be assumed that the creep feed intake varied among piglets and some of them did not eat any creep feed. Therefore, serum glucose levels may also have indicated an upregulation of hepatic gluconeogenesis, allowing the piglet to produce glucose endogenously (Girard et al., 1992). The present serum triglyceride and cholesterol levels may support increased lipogenesis and cholesterogenesis during the suckling phase (Titchenell et al., 2017).

Concentrations of SCFA in gastric and large intestinal digesta developed differently during the suckling phase. The greater stomach size per kg of body weight and the higher gastric SCFA concentration (mainly acetate) on DoL3 supported the role of the stomach as storage organ and the importance of microbial activity for the neonatal upper digestive tract (Metzler-Zebeli et al., 2022; Gormley et al., 2024). Due to the longer retention in the stomach, it is reasonable to assume that less substrate entered the large intestine on DoL3, explaining the largely lower colonic SCFA concentrations on DoL3 compared to DoL7 in colonic digesta. The higher gastric acetate concentration on DoL3 may have been due to the predominance of *Limosilactobacillus* and *Streptococcus* in gastric digesta (Yosi et al., 2024), which comprise heterofermentative species (Gobbetti and Calasso, 2014; Yang et al., 2022). From DoL7, *Lactobacillus* became the dominating genus in gastric digesta (Yosi et al., 2024), probably shifting the fermentation from acetate to lactate, which we did not measure in the present study. Accordingly, age-related expression of *FFAR2* and *FFAR3* in the jejunum during the suckling phase probably reflected the SCFA present in the lumen, with acetate being the major FA. Free fatty acid receptor-2 and -3 have equal affinity to acetate, whereas FFAR-2 has a higher affinity for propionate and butyrate (Kaiko and Stappenbeck, 2014; McKenzie et al., 2017). Likewise, lactate-sensing receptors *FFAR1* and *HCAR1* were higher expressed on DoL3 and/or 7 compared to the later DoL, indicating the importance of lactate for gut development and metabolism. Even though the higher milk intake with increasing age should have stimulated fermentation along the gastrointestinal tract, which is supported by cecal and colonic SCFA concentrations, jejunal and cecal expression of certain FA receptors and transporters were downregulated with age. An explanation may be that during the first DoL, the intestinal mucosa sought FA to promote development and adjusted receptor and transporter expression levels according to luminal FA availability. This may also explain the age-related expression patterns of the long-chain FA transporter *FFAR1* and bile acid receptor, being higher at the jejunal and cecal mucosa on DoL3 than 28. Bile acid production increases from birth to weaning (Metzler-Zebeli et al., 2023). Consequently, stronger sensing of bile acids via *FXR* in the first DoL with lower bile acid concentrations in digesta may be important for the development of lipid homeostasis through the gut-liver axis. Conversely, the present age-related development of FA and bile acid receptor and transporter expression diverged from previous findings in creep-fed piglets (Lerch et al., 2023), supporting our assumption of the role of the local gut microbial activity for the gut mucosal response but probably also the influence of other factors, such as the actual feed intake.

The jejunal and cecal expression related to mucosal barrier, defense and antioxidative properties displayed gut-site specific developmental patterns from DoL3 to 28. Genes within the proinflammatory signaling cascade were less expressed in the jejunum compared to the cecum, which may be linked to lower immune cell populations and/or lower microbial diversity and quantitative abundance in the upper gut compared to the cecum (Arnaud et al., 2020; Yosi et al., 2024). We expected a decreasing PRR expression along the gut as result of the development of immunological tolerance. However, jejunal PRR expression did not show the corresponding downregulation with increasing age of the piglets. By contrast, jejunal expression of certain PRR (ie, *TLR7*, *TLR8*, *TLR9*, and *NOD2*) increased from DoL3 to 28, reflecting maturation-related changes in the composition of the microbiota and/or increasing abundance of their microbial antigens (Kawasaki and Kawai, 2014). Accordingly, the bacterial and fungal abundances in cecal digesta of the present piglets showed great fluctuations from DoL3 to 28 (Yosi et al., 2024), triggering different PRR expressions. Accordingly, the developing cecal microbiota community on DoL3 appeared to be richer in flagellin, zymosan, peptidoglycan, D-glytamyl-meso-diamino-pimelic acid, and muramyl dipeptide due to the higher expression of *TLR1*, *TLR2*, *TLR5*, *NOD1*, and *NOD2* on DoL3 compared to the later time points (Kawasaki and Kawai, 2014). Upon activation, PRR trigger signal transduction pathways that culminate in the activation of nuclear transcription factors, such as NF-kB, to regulate the expression of cytokines (Kawasaki and Kawai, 2014). While proinflammatory signaling via NF-kB appeared to decrease in the jejunum with age, as indicated by the lower expression of *NKAP*, different signaling routes were triggered more strongly, leading to an upregulated *TNFA* expression from DoL3 to 28. Correlation analysis supported a respective association that signaling via *TLR4*, *TLR7*, *TLR8*, *TRL9*, and *NOD2* may have contributed to the upregulated *TNFA* expression with age. Simultaneously, expression levels of anti-inflammatory factors, such as *IL10* and *TGFB1*, increased in the jejunum and cecum from DoL3 to 28, which likely reduced the inflammatory response at the gut mucosa and contributed to the build-up of immune tolerance. The moderate positive correlations between these factors and expression of most PRR in the jejunum and to a lesser extent in the cecum would support this assumption. Correlation analysis may further support the assumption that proinflammatory signaling pathways affected the expression of genes for epithelial barrier function by modulating the expression of mucins and tight-junction proteins (Takano et al., 2014). However, correlation patterns were gut site specific. Overall, there was a general trend for a reduced expression of genes related to barrier function and antioxidative activity with increasing age of the piglet, which may further support the build-up of immunological tolerance.

Although piglets were creep fed, their average solid feed intake remained low in the suckling period (Yosi et al., 2024). This let us assume that their solid feed intake immediately after weaning remained low, leading to lower serum glucose and triglyceride levels on DoL31 and 35 compared to DoL28. In the case of triglycerides, they also reflect the change in the main energy substrate from high-fat milk to solid, starch-rich feed. The negative consequences of the lower feed intake after weaning for gastrointestinal SCFA was more obvious in gastric and cecal digesta than in colonic digesta, corresponding to the drastic changes in the local bacterial and fungal communities due to the change in diet (Yosi et al., 2024). By contrast, mid-colonic fermentation was sustained and concentrations even increased postweaning, which may have been caused by a considerably slower digesta passage along the colon due to reduced vagus nerve stimulation (Atanassova et al., 1991). Certain but not all receptors and transporters for luminal FA in the jejunum and cecum may have responded to the decreased luminal availability of their ligands, which may be supported by the present correlations (e.g., between acetate concentration in cecal digesta and downregulation of *FFAR3* expression postweaning). Despite piglets eating less directly after weaning, bile acid signaling in the jejunum, as indicated by the *FXR* expression, seemed to be almost similar on DoL31 compared to DoL28 as also reported by Lerch et al. (2023). Nevertheless, there was a general trend in the cecum for decreasing *FXR* expression after weaning in the present study, which was different to our previous results (Lerch et al., 2023) and may be explained by different microbial metabolism of bile acids (Yang et al., 2025) between the two studies. In contrast to the reported upregulated proinflammatory response via TLR-4/IL-1β one to four days after weaning in previous research (Tao et al., 2015; Tang et al., 2021; Pié et al., 2024) but in line with our previous research (Lerch et al., 2023), the present gene expression data did not support an increased proinflammatory signaling in the jejunum and cecum on DoL31 as PRR were less expressed at both gut sites. If lower PRR activation resulted in lower inflammatory signaling, we would have expected negative correlations with barrier function and mucosal secretions. However, Pearson correlations showed the opposite. Consequently, the downregulated jejunal expression of anti-inflammatory cytokines (*IL10* and *TGFB1*), antioxidative enzyme (*GPX1*), antimicrobial secretion (*IAP*) and barrier function (*CLDN4*) on DoL31 compared to DoL28 might suggest a disturbance in the mucosal homeostasis from DoL28 to 31. Similarly, the higher expression of proinflammatory *IL1B* and *IL6* may have triggered a weakening of the mucosal barrier after weaning as indicated by the lower expression of *MUC2*, *OCLN*, and *ZO1* on DoL31 compared to DoL28. However, in contrast to the jejunal expression, the cecal expression levels of these genes were similar on DoL35 compared to DoL31. Therefore, it is thinkable that their cecal expression levels together with the lower expression of certain PRR (ie, *TLR2*, *TLR5*, and *TLR6*) on DoL31 already represented a novel homeostatic state after weaning.

In conclusion, the present results for the neonatal development of serum parameters and gut mucosal sensing of FA and bile acids, antioxidative capacities, barrier function, and innate immune response represent a referable source for future studies and clinical diagnostics. The results confirm study-specific, age-related expression patterns of genes associated with mucosal metabolite sensing or defense mechanisms in the jejunum and cecum from birth to weaning and thereafter. Contrary to our assumption, we did not observe a strong upregulation of proinflammatory signals three days after weaning.

## Supplementary Material

skaf313_Supplementary_Data

## Abbreviations:

ACTG: γ-actin
ALP: alkaline phosphatase
ALT: alanine transaminase
AST: aspartate transaminase
B2M: β2-microglobulin
cDNA: complementary DNA
CLDN: claudin
DoL: day of life
FA: fatty acid
FFAR: free fatty acid receptor
FXR: farnesoid X receptor
GAPDH: glyceraldehyde 3-phosphate-dehydrogenase
GPX: glutathione peroxidase
HCAR: hydroxycarboxylic acid receptor
HPRT: hypoxanthine phosphoribosyl transferase
IAP: intestinal alkaline phosphatase
IL: interleukin
MCT: monocarboxylate transporter
MUC: mucin
NKAP: nuclear factor kappa B-activating protein
NOD: nucleotide-binding oligomerization domain
OAZ1: ornithine decarboxylase antizyme 1
OCLN: occludin
PRR: pattern recognition receptor
SCFA: short-chain fatty acids
SEM: standard errors of the mean
SMCT: sodium-coupled monocarboxylate transporter
SOD: superoxide dismutase
TGFB: transforming growth factor beta
TLR: toll-like receptor
TNFA: tumor necrosis factor alpha
ZO1: zonula occludens
