# Supplementary material for: Age-related evolution of serum biochemistry and intestinal fatty acid signaling, innate immune response, and barrier function in suckling and newly weaned piglets
Source: J Anim Sci. 2025 Sep 8;103:skaf313. doi: 10.1093/jas/skaf313 (PMC12510401; doi:10.1093/jas/skaf313)
Supplement: skaf313_Supplementary_Data [file skaf313_supplementary_data.docx]

Running title: Age-related gut development

**Age-related evolution of serum biochemistry and intestinal fatty acid signaling, innate immune response and barrier function in suckling and newly weaned piglets**

Fitra Yosi^*,#,†^, Kristina Hartinger^*,#^, Frederike Lerch^*,#^, Julia C. Vötterl^*,#^, Simone Koger^#,§^, Suchitra Sharma^#,§^, Doris Verhovsek^¶^, and Barbara U. Metzler-Zebeli^*,#,1^

^*^Centre for Veterinary Systems Transformation and Sustainability, Clinical Department for Farm Animals and Food System Science, University of Veterinary Medicine Vienna, Vienna 1210, Austria.

^#^Christian-Doppler Laboratory for Innovative Gut Health Concepts of Livestock, Institute of Animal Nutrition and Functional Plant Compounds, Centre for Animal Nutrition and Welfare, University of Veterinary Medicine Vienna, Vienna 1210, Austria.

^†^Department of Animal Science, Faculty of Agriculture, University of Sriwijaya, Palembang 30662, Indonesia.

^§^Centre for Animal Nutrition and Welfare, Clinical Department for Farm Animals and Food System Science, University of Veterinary Medicine Vienna, Vienna 1210, Austria.

^¶^Clinical Centre for Population Medicine in Fish, Pig and Poultry, Clinical Department for Farm Animals and Food System Science, University of Veterinary Medicine Vienna, Vienna 1210, Austria.

^1^Corresponding author: [barbara.metzler@vetmeduni.ac.at](mailto:barbara.metzler@vetmeduni.ac.at)

**Supplementary Materials**

Table S1. Ingredient composition of prestarter diets (adapted from Yosi et al., 2024)

| Ingredients, % | Prestarter^1^ |
| --- | --- |
| Barley meal | 29.7 |
| Corn meal | 10.8 |
| Wheat meal | 10.0 |
| Fullfat soy | 14.1 |
| Wheat pressure cooked | 9.9 |
| Sweet whey powder | 3.0 |
| Potato protein | 5.0 |
| Lignocellulose | 0.9 |
| Palm kernel | 2.0 |
| Dried vinasse^4^ | 1.9 |
| Dextrose | 5.0 |
| Lactose | 3.0 |
| Rapeseed oil | 0.5 |
| Lysine HCl | 0.7 |
| Threonine | 0.3 |
| Methionine | 0.3 |
| Tryptophane | 0.1 |
| Limestone (calcium carbonate) | 0.6 |
| Sodium chloride | 0.5 |
| Mono calcium phosphate | 1.4 |
| Magnesium phosphate | 0.2 |
| Vitamin/trace element premix | 0.4 |

^1^Vitamin and mineral composition per kg feed: 16,000 IE of vitamin A, 2,000 IE of vitamin D3, 150 mg of vitamin E, 4.0 mg of vitamin K3, 2.8 mg of vitamin B1, 8.2 mg of vitamin B2, 5.0 mg of vitamin B6, 50 mg of vitamin B12, 60 mg of nicotinic acid, 20 mg of panthothenic acid, 500 mg of cholin chloride, 1,050 mcg of folic acid, 150 mcg of biotin, 124 mg of Fe as iron(II) sulfat monohydrate, 80 mg of Mn as manganese(II) oxide, 3.1 mg of I as calcium iodate, 121 mg of Zn as zinc oxide, 0.45 mg of Se as sodium selenite, 124 mg of Cu as copper(II) sulfat pentahydrate. Technological additives: 250 FTU of phytase (4a16), 100 mg of butylated hydroxytoluene.

^4^CITROFEED, dried residues from citric acid production.

**Table S2**. Analyzed nutrient composition of the milk replacer and prestarter diet for piglets (adapted from Yosi et al., 2024)

| Chemical composition, % dry matter | Milk replacer^1,2^ | Prestarter diet^2^ |
| --- | --- | --- |
| Dry matter, % | 91.6 | 89.8 |
| Crude protein | 23.3 | 18.2 |
| Crude fiber | 1.9 | 4.9 |
| Ether extract | 10.3 | 7.3 |
| Crude ash | 6.8 | 5.1 |
| Nitrogen-free extract | 57.9 | 65.0 |
| Starch | 25.4 | 42.5 |
| Metabolizable energy, MJ/kg | 16.9 | 15.4 |
| Macro minerals, % |  |  |
| Calcium | 0.80 | 0.73 |
| Phosphorus | 0.80 | 0.72 |
| Magnesium | 0.20 | 0.21 |
| Potassium | 1.09 | 0.67 |
| Sodium | 0.50 | 0.24 |
| Trace minerals, ppm |  |  |
| Iron | 264 | 333 |
| Manganese | 87 | 118 |
| Zinc | 129 | 182 |
| Copper | 146 | 152 |

^1^NuriStart Sweet, BIOMIN Holding GmbH, Part of dsm-firmenich, Getzersdorf, Austria. Ingredients: Wheat flour, whey protein concentrate, whey powder, extruded soybeans, puffed corn, rolled oats, soy protein concentrate, sugar, dextrose, palm oil, monocalcium phosphate,

coconut oil, sodium chloride, calcium carbonate, and salmon oil. Technological additives per kg: 400 mg sepiolite (E562), 3,000 mg citric acid (E330), 0.07 mg propyl gallate (E310), 1 mg butyrylhydroxy toluol (E321). Zootechnical additives per kg: 200 FXU endo-1,4-beta-xylanase EC 3.2.1.8; 1,000 FYT 6-phytase EC 3.1.3.26. Nutritional additives per kg: 16,000 IE of vitamin A, 2,000 IE of vitamin D3, 150 mg vitamin E, 200 mg vitamin C, 195 mg Fe as iron (II) sulfate monohydrate, 2.0 mg I as calcium iodate anhydrous, 0.40 mg Se as sodium selenite, 60 mg Mn as manganese (II) oxide, 140 mg Cu as copper (II) sulfate pentahydrate, 100 mg Zn as zinc oxide.

^2^Piglets were fed with 100% milk replacer from day of life (DoL) 3 to 23; combinations milk replacer and prestarter diet with a ratio of 70:30 (w/w) on DoL24, 50:50 (w/w) on DoL25, and 30:70 (w/w) on DoL26, respectively; and 100% prestarter diet on DoL27 and 28.

**Table S3.** Oligonucleotide primers used for quantitative PCR to assess jejunal and cecal gene expression in suckling and newly weaned piglets

| Genes^1^ | Accession number^2^ | Primer sequence (5’ to 3’)^3^ | | R^2^ | Efficiency, % | Amplicon size, bp | Reference |
| --- | --- | --- | --- | --- | --- | --- | --- |
| Reference genes | | | | | | | |
| *ACTG* | XM_003357928.4 | F: | GGGCATCCTGACCCTCAAG | 0.997 | 101 | 89 | (Klinsoda et al., 2020) |
|  |  | R: | TGTAGAAGGTGTGATGCCAGATCT |  |  |  |  |
| *B2M* | NM_213978.1 | F: | CCCCCGAAGGTTCAGGTT | 0.999 | 98 | 66 | (Metzler-Zebeli et al., 2017) |
|  |  | R: | GCAGTTCAGGTAATTTGGCTTTC |  |  |  |  |
| *GAPDH* | NM_001206359.1 | F: | GGCGTGAACCATGAGAAGTATG | 0.998 | 104 | 60 | (Metzler-Zebeli et al., 2017) |
|  |  | R: | GGTGCAGGAGGCATTGCT |  |  |  |  |
| *HPRT* | NM_001032376.2 | F: | AGAAAAGTAAGCAGTCAGTTTCATATCAGT | 0.997 | 98 | 131 | (Metzler-Zebeli et al., 2017) |
|  |  | R: | ATCTGAACAAGAGAGAAAATACAGTCAATAG |  |  |  |  |
| *OAZ1* | NM_001122994.2 | F: | TCGGCTGAATGTAACAGAGGAA | 0.997 | 97 | 70 | (Metzler-Zebeli et al., 2017) |
|  |  | R: | GAGCCTGGATTGGACGTTTAAA |  |  |  |  |
| Fatty acid receptors and transporters, bile acid receptor | | | | | | | |
| *FFAR1* | XM_013998289.2 | F: | ACTTAGGGAAAGAACTGAGCCT | 0.999 | 99 | 105 | (Lerch et al., 2023) |
|  |  | R: | GATGAAATGCGGCAGCTTACC |  |  |  |  |
| *FFAR2* | NM_001278758.1 | F: | CTGCCTGGGATCGTCTGTG | 1.000 | 97 | 249 | (Newman et al., 2018) |
|  |  | R: | CATACCCTCGGCCTTCTGG |  |  |  |  |
| *FFAR3* | NM_001315601.1 | F: | GCCCTTGCCCTTCATCTTCT | 1.000 | 98 | 136 | (Newman et al., 2018) |
|  |  | R: | CCGGGTCTTGTACCAGAGTG |  |  |  |  |
| *FFAR4* | NM_001204766.2 | F: | GCACCCGTGTACCTGCTTTA | 1.000 | 99 | 127 | (Lerch et al., 2023) |
|  |  | R: | AAGGAACCCACAGCAAATCCTTT |  |  |  |  |
| *HCAR1* | NM_001145381.1 | F: | AATGCCATCTCCAACCGGAC | 0.999 | 98 | 153 | (Vötterl et al., 2023) |
|  |  | R: | GCCATTGGCTGACTCCATGA |  |  |  |  |
| *MCT1* | AM286425.1 | F: | GGTGGAGGTCCTATCAGCAG | 0.999 | 102 | 74 | (Metzler-Zebeli et al., 2015) |
|  |  | R: | AAGCAGCCGCCAATAATCAT |  |  |  |  |
| *SMCT1* | NM_001291414.1 | F: | AATCCTCACCTGCTCAGTGC | 0.994 | 99 | 172 | (Metzler-Zebeli et al., 2022) |
|  |  | R: | GTAAGCGCAGGCCACAAAAA |  |  |  |  |
| *SMCT2* | XM_003122908.4 | F: | TGGGACACAACTTTCCCTTGG | 0.995 | 99 | 407 | (Metzler-Zebeli et al., 2022) |
|  |  | R: | GGAAGGTGGTTCTCCTGTGG |  |  |  |  |
| *FXR* | KF597010.1 | F: | AAGCCTGCCAAAGGTGTACT | 1.000 | 99 | 156 | (Vötterl et al., 2023) |
|  |  | R: | GGGGTAGAAACCCAGGTTGG |  |  |  |  |
| Pattern recognition receptors | | | | | | | |
| *TLR1* | AB086376.1, NM_001031775.1 | F: | TTTGCCCACCACAACCTCTT | 0.999 | 100 | 153 | (Vötterl et al., 2023) |
|  |  | R: | GCTCTTCTCCTTGGGCCATT |  |  |  |  |
| *TLR2* | NM_213761.1 | F: | AATAAGTTGAAGACGCTCCCAGAT | 1.000 | 100 | 97 | (Metzler-Zebeli et al., 2015) |
|  |  | R: | GTTGCTCCTTAGAGAAAGTATTGATCGT |  |  |  |  |
| *TLR4* | AB188301.2 | F: | TGTGGCCATCGCTGCTAAC | 0.998 | 101 | 124 | (Metzler-Zebeli et al., 2015) |
|  |  | R: | GGTCTGGGCAATCTCATACTCA |  |  |  |  |
| *TLR5* | NM_001348771.1 | F: | GGCCAGAGGGCTATCTATCGT | 0.996 | 99 | 104 | (Arnaud et al., 2020) |
|  |  | R: | GTGACCGTCCTGATGTAGTTGAAG |  |  |  |  |
| *TLR6* | NM_213760.2 | F: | GCCCTGGTATCTCAGGATGC | 0.996 | 100 | 134 | (Vötterl et al., 2023) |
|  |  | R: | CCCAGGCAGAATCGTGTTCA |  |  |  |  |
| *TLR7* | NM_001097434.1 | F: | CCAACAAGTGGTTGCTGCTC | 0.998 | 101 | 105 | (Vötterl et al., 2023) |
|  |  | R: | CATCGATGGGCAGAGATTTTTC |  |  |  |  |
| *TLR8* | AB092975 | F: | AAGACCACCACCAACTTAGCC | 1.000 | 101 | 105 | (Arnaud et al., 2020) |
|  |  | R: | GACCCTCAGATTCTCATCCATCC |  |  |  |  |
| *TLR9* | AY859728 | F: | CACGACAGCCGAATAGCAC | 0.999 | 98 | 122 | (Arnaud et al., 2020) |
|  |  | R: | GGGAACAGGGAGCAGAGC |  |  |  |  |
| *NOD1* | NM_001114277.1 | F: | CTAACCATGGAAAAGCAGGGC | 1.000 | 104 | 96 | (Vötterl et al., 2023) |
|  |  | R: | CAGATGCTCCCGGTTGACTT |  |  |  |  |
| *NOD2* | AB195466.1 | F: | GAGCGCATCCTCTTAACTTTC | 0.999 | 99 | 66 | (Arnaud et al., 2020) |
|  |  | R: | ACGCTCGTGATCCGTGAAC |  |  |  |  |
| Transcription factor, cytokines, and antioxidative enzymes | | | | | | | |
| *NKAP* | XR_002340533.1 | F: | TGGACATTCAACGTCAACAGC | 0.998 | 100 | 75 | (Metzler-Zebeli et al., 2022) |
|  |  | R: | AGTGAGCTCAGCATTTCATCCAT |  |  |  |  |
| *TNFA* | NM_214022.1 | F: | GCCCTTCCACCAACGTTTTC | 1.000 | 101 | 97 | (Vötterl et al., 2023) |
|  |  | R: | CAAGGGCTCTTGATGGCAGA |  |  |  |  |
| *IL1B* | NM_214055.1 | F: | CTACCCTCTCCAGCCAGTCT | 0.996 | 101 | 155 | (Vötterl et al., 2023) |
|  |  | R: | GGGTGCAGCACTTCATCTCT |  |  |  |  |
| *IL6* | M86722.1 | F: | CGCAGCCTTGAGGATTTCC | 0.998 | 99 | 67 | (Metzler-Zebeli et al., 2018) |
|  |  | R: | TCAGGTGCCCCAGCTACATT |  |  |  |  |
| *IL10* | NM_214041.1 | F: | GCCTACATGACGATGAAGATGA | 0.999 | 99 | 112 | (Metzler-Zebeli et al., 2018) |
|  |  | R: | TGAAAGTCTCCAATTTGTATCCTAGAGT |  |  |  |  |
| *TGFB1* | NM_214015.2 | F: | ATCTCGCCCATCTCGGTTT | 0.999 | 99 | 100 | (Metzler-Zebeli et al., 2018) |
|  |  | R: | AAGTTGAGGCTCTCAGGGAGAA |  |  |  |  |
| *SOD1* | NM_001190422.1, GQ913661.1 | F: | ACAGTGTTAGTAACGGGAACCA | 0.997 | 98 | 152 | (Vötterl et al., 2023) |
|  |  | R: | TCTTGATCCTTTGGCCCACC |  |  |  |  |
| *GPX1* | NM_214201.1, KJ686126.1 | F: | CTAGCAGTGCCTAGAGTGCC | 0.996 | 100 | 142 | (Vötterl et al., 2023) |
|  |  | R: | CGCCCATCTCAGGGGATTTT |  |  |  |  |
| First line of defense genes | | | | | | | |
| *MUC2* | XM_021082584.1 | F: | GCTCCAGAGAGAAGGCAGAA | 0.998 | 100 | 162 | (Vötterl et al., 2023) |
|  |  | R: | ACAGCGAACTCCTTGTAGGC |  |  |  |  |
| *MUC4* | DQ848681.1 | F: | GAGCAGAGCCCTGAGGGTA | 0.999 | 99 | 101 | (Vötterl et al., 2023) |
|  |  | R: | CCCTGGAACCAGAGCTTCAG |  |  |  |  |
| *CLDN1* | NM_001244539.1 | F: | TGATGAGGTGCAGAAGATGC | 0.999 | 98 | 88 | (Klinsoda et al., 2020) |
|  |  | R: | CCATGCTGTGGCAACTAAGA |  |  |  |  |
| *CLDN4* | NM_001161637.1 | F: | CAACTGCGTGGATGATGAGA | 1.000 | 100 | 140 | (Klinsoda et al., 2020) |
|  |  | R: | CCAGGGGATTGTAGAAGTCG |  |  |  |  |
| *OCLN* | NM_001163647.2 | F: | TTGTGGGACAAGGAACGTATTTA | 0.999 | 100 | 76 | (Metzler-Zebeli et al., 2015) |
|  |  | R: | TGCCTGCCGACACGTTT |  |  |  |  |
| *ZO1* | AJ318101.1 | F: | TCAAGGTCTGCCGAGACAAC | 0.999 | 99 | 75 | (Metzler-Zebeli et al., 2022) |
|  |  | R: | CCAAAGGACTCAGCAGGGTT |  |  |  |  |
| *IAP* | XM_003133729.4 | F: | AGGAACCCAGAGGGACCATTC | 0.999 | 98 | 83 | (Metzler-Zebeli et al., 2018) |
|  |  | R: | CACAGTGGCTGAGGGACTTAGG |  |  |  |  |

^1^*ACTG*, γ-actin; *B2M*, β2-microglobulin; *GAPDH*, glyceraldehyde-3-phosphate-dehydrogenase; *HPRT*, hypoxanthine phosphoribosyl transferase; *OAZ1*, ornithine decarboxylase antizyme 1; *FFAR1*, free fatty acid receptor 1; *FFAR2*, free fatty acid receptor 2; *FFAR3*, free fatty acid receptor 3; *FFAR4*, free fatty acid receptor 4; *HCAR1*, hydroxycarboxylic acid receptor 1; *MCT1*, monocarboxylate transporter 1; *SMCT1*, sodium coupled monocarboxylate transporter 1; *SMCT2*, sodium coupled monocarboxylate transporter 2; *FXR*, farnesoid X receptor; *TLR1*, toll-like receptor 1; *TLR2*, toll-like receptor 2; *TLR4*, toll-like receptor 4; *TLR5*, toll-like receptor 5; *TLR6*, toll-like receptor 6; *TLR7*, toll-like receptor 7; *TLR8*, toll-like receptor 8; *TLR9*, toll-like receptor 9; *NOD1*, nucleotide-binding oligomerization domain 1; *NOD2*, nucleotide-binding oligomerization domain 2; *NKAP,* NFKB activating protein; *TNFA*, tumor necrosis factor alpha; *IL1B*, interleukin 1 beta; *IL6*, interleukin 6; *IL10*, interleukin 10; *TGFB1*, transforming growth factor beta 1; *SOD1*, superoxide dismutase 1; *GPX1*, glutathione peroxidase 1; *MUC2*, mucin 2; *MUC4*, mucin 4; *CLDN1*, claudin 1; *CLDN4*, claudin 4; *OCLN*, occludin; *ZO1*, zonula occludens 1; *IAP*, intestinal alkaline phosphatase.

^2^National Center for Biotechnology Information (NCBI; http://www.ncbi.nlm.nih.gov/sites/entrez?db=gene).

^3^F, forward primer; R, reverse primer.

**Table S4.** Descriptive statistics for average daily creep feed intake of piglets during the suckling period (adapted from Yosi et al., 2024)

| Average daily feed intake, g/DM^1,2^ | Mean | SE |
| --- | --- | --- |
| DoL3 to 9 | 19.7 | 1.14 |
| DoL10 to 16 | 16.5 | 0.77 |
| DoL17 to 23 | 20.9 | 0.88 |
| DoL24 to 26 | 23.5 | 1.79 |
| DoL27 to 28 | 17.4 | 1.92 |

DM, dry matter; DoL, day of life; SE, standard error.

^1^Creep feed intake was estimated on litter basis.

^2^Piglets were fed with 100% milk replacer from DoL3 to 23; combinations of milk replacer and prestarter diet with a ratio of 70:30 (w/w) on DoL24, 50:50 (w/w) on DoL25, and 30:70 (w/w) on DoL26, respectively; and 100% prestarter diet on DoL27 and 28.

**Literature cited**

Arnaud, A. P., V. Rome, M. Richard, M. Formal, S. David‐Le Gall, and G. Boudry. 2020. Post‐natal co‐development of the microbiota and gut barrier function follows different paths in the small and large intestine in piglets. FASEB J. 34:1430–1446. doi:10.1096/fj.201902514R.

Klinsoda, J., J. Vötterl, Q. Zebeli, and B. U. Metzler-Zebeli. 2020. Alterations of the viable ileal microbiota of the gut mucosa-lymph node axis in pigs fed phytase and lactic acid-treated cereals. Appl. Environ. Microbiol. 86. doi:10.1128/AEM.02128-19.

Lerch, F., F. Yosi, J. C. Vötterl, S. Koger, J. Ehmig, S. Sharma, D. Verhovsek, and B. U. Metzler-Zebeli. 2023. An insight into the temporal dynamics in the gut microbiome, metabolite signaling, immune response, and barrier function in suckling and weaned piglets under production conditions. Front. Vet. Sci. 10. doi:10.3389/fvets.2023.1184277.

Metzler-Zebeli, B. U., E. Mann, R. Ertl, S. Schmitz-Esser, M. Wagner, D. Klein, M. Ritzmann, and Q. Zebeli. 2015. Dietary calcium concentration and cereals differentially affect mineral balance and tight junction proteins expression in jejunum of weaned pigs. Br. J. Nutr. 113:1019–1031. doi:10.1017/S0007114515000380.

Metzler-Zebeli, B. U., R. Ertl, D. Grüll, T. Molnar, and Q. Zebeli. 2017. Enzymatically modified starch up-regulates expression of incretins and sodium-coupled monocarboxylate transporter in jejunum of growing pigs. Animal. 11:1180–1188. doi:10.1017/S1751731116002615.

Metzler-Zebeli, B. U., M. A. Newman, D. Grüll, and Q. Zebeli. 2018. Consumption of transglycosylated starch down-regulates expression of mucosal innate immune response genes in the large intestine using a pig model. Br. J. Nutr. 119:1366–1377. doi:10.1017/S0007114518001113.

Metzler-Zebeli, B. U., S. Koger, S. Sharma, A. Sener-Aydemir, U. Ruczizka, H. Kreutzmann, and A. Ladinig. 2022. Short-chain fatty acids modulate permeability, motility and gene expression in the porcine fetal jejunum ex vivo. Nutrients. 14:2524. doi:10.3390/nu14122524.

Newman, M. A., R. M. Petri, D. Grüll, Q. Zebeli, and B. U. Metzler-Zebeli. 2018. Transglycosylated starch modulates the gut microbiome and expression of genes related to lipid synthesis in liver and adipose tissue of pigs. Front. Microbiol. 9:224. doi:10.3389/fmicb.2018.00224.

Vötterl, J. C., F. Lerch, H. E. Schwartz-Zimmermann, E. L. Sassu, L. Schwarz, R. Renzhammer, M. Bünger, S. Koger, S. Sharma, A. Sener-Aydemir, N. M. Quijada, E. Selberherr, F. Berthiller, and B. U. Metzler-Zebeli. 2023. Plant-oriented microbiome inoculum modulates age-related maturation of gut-mucosal expression of innate immune and barrier function genes in suckling and weaned piglets. J. Anim. Sci. 101. doi:10.1093/jas/skad165.

Yosi, F., F. Lerch, J. C. Vötterl, S. Koger, D. Verhovsek, and B. U. Metzler-Zebeli BU. 2024. Lactation-related dynamics of bacterial and fungal microbiomes in feces of sows and gut colonization in suckling and newly weaned piglets. J. Anim. Sci. 102:skae321. doi: 10.1093/jas/skae321.
